# The interplay between genetic and bioelectrical signaling permits a spatial regionalisation of membrane potentials in model multicellular ensembles

**DOI:** 10.1038/srep35201

**Published:** 2016-10-12

**Authors:** Javier Cervera, Salvador Meseguer, Salvador Mafe

**Affiliations:** 1 Dept. de Termodinàmica, Facultat de Física, Universitat de València, E-46100 Burjassot, Spain; 2 Laboratory of RNA Modification and Mitochondrial Diseases, Centro de Investigación Príncipe Felipe, Valencia 46012, Spain

## Abstract

The single cell-centred approach emphasises ion channels as specific proteins that determine individual properties, disregarding their contribution to multicellular outcomes. We simulate the interplay between genetic and bioelectrical signals in non-excitable cells from the local single-cell level to the long range multicellular ensemble. The single-cell genetic regulation is based on mean-field kinetic equations involving the mRNA and protein concentrations. The transcription rate factor is assumed to depend on the absolute value of the cell potential, which is dictated by the voltage-gated cell ion channels and the intercellular gap junctions. The interplay between genetic and electrical signals may allow translating single-cell states into multicellular states which provide spatio-temporal information. The model results have clear implications for biological processes: (*i*) bioelectric signals can override slightly different genetic pre-patterns; (*ii*) ensembles of cells initially at the same potential can undergo an electrical regionalisation because of persistent genetic differences between adjacent spatial regions; and (*iii*) shifts in the normal cell electrical balance could trigger significant changes in the genetic regulation.

The multicellular patterns characteristic of processes such as morphogenesis, regeneration and carcinogenesis have traditionally been described on the basis of chemical signals and concentrations gradients. However, the transport of the ions and signalling molecules which affect downstream biochemical cascades and transcriptional processes is also influenced by the electrical cell state and intercellular coupling^1^,^2^,^3^,^4^,^5^,^6^,^7^,^8^,^9^,^10^,^11^. The purpose of this study is to propose a highly idealised model for the integration of genetic expression and bioelectrical signals in the case of non-excitable cells, from the single-cell level to the multicellular ensemble.

Genetic networks influence and in turn may be influenced by bioelectrical signals. The proteins forming the ion channels in the cell membrane and the intercellular gap junctions regulate the bioelectric signals. These signals influence the genetic pathways via the unidirectional transport and spatial accumulation of calcium and different signaling molecules, electrically-induced conformational changes in the voltage-sensing domains of membrane proteins, and the activation of voltage-gated channels^1^,^2^,^3^,^4^,^5^,^6^. In turn, these proteins are regulated by the genetic and epigenetic networks that allow transcriptional control^7^,^8^. This bidirectional flow is not simply described by specifying the protein cellular levels because ion channels and gap junctions are gated post-translationally by the particular dynamics of the cell environment^1^,^7^.

The interplay between genetic and bioelectrical signals is relevant to multicellular patterning. In *Xenopus* embryogenesis, the spatial regionalisation of cell electrical potentials specifies a prepattern for craniofacial patterning genes^9^,^10^. The disruption of the normal regionalisation of potentials can affect the expression patterns of genes critical for the patterning of the face^10^. Other mechanisms that transduce alterations of the cell bioelectrical states into downstream transcriptional changes include the transporters of calcium and signaling molecules (serotonin, butyrate) which are regulated by cell membrane potentials^1^. In particular, the voltage-gated sodium^11^, potassium^5^, and calcium^2^ channels are known to influence gene regulation and cancer. The potential biomedical opportunities of reprogramming cells and tissue patterning via bioelectrical pathways have recently been reviewed^1^.

The dominant biochemical approach emphasises reaction-diffusion processes^12^,^13^ and disregards often the bioelectrical signals that influence the single-cell cycle and may provide positional information in embryogenesis, regeneration and cancer^1^,^7^,^14^,^15^,^16^,^17^,^18^. We have developed recently a simple theoretical scheme for describing multicellular ensembles using basic concepts from membrane and ion channel biophysics^19^. The model ignores diffusion-reaction processes^20^,^21^,^22^ by focussing on collective electrical states and multicellular patterning. We consider now the case of *voltage-dependent genetic regulation*, from the local single-cell level to the long range multicellular ensemble. To this end, we study the electrically-induced changes in genetic regulation using a network of intercellular gap junctions working as non-linear bioelectrical units.

The single-cell genetic regulation considered here is based on mean-field kinetic equations for the mRNA and protein relative concentrations. The transcription is assumed to depend on the cell potential, which is modulated by the voltage-gated cell ion channels and the intercellular gap junctions. We consider simple physical mechanisms to translate single-cell bioelectric states into multicellular regionalisations that can be observed using voltage-sensitive dyes^23^,^24^,^25^. While the importance of chemical signals and concentrations gradients in the genetic regulation and establishment of biological patterns is well documented^12^,^13^,^21^,^26^, the regionalisation of single-cell polarisations is not well understood and contributes to the spatial distribution of signalling molecules in the ensemble^1^,^4^,^27^,^28^.

## Experimental Basis

The cell resting potential is usually defined as the negative potential difference that exists between the cell cytoplasm and the extracellular environment at zero total current^19^,^29^. Differentiated cells tend to be more polarised, showing a high absolute value of the negative cell potential, whereas proliferating cells (embryonic, stem and tumor cells) are depolarised, showing a low absolute value of this potential^1^,^7^,^17^,^18^. However, the spatio-temporal maps of electrical potential in multicellular systems should be regarded as an ensemble characteristic rather than a simple aggregate of single-cell values^19^,^30^.

Bioelectrical potentials are involved in the intercellular communication and collective information processing required for multicellular processes such as developmental patterning and carcinogenesis^1^,^31^. Experimentally, these potentials can be resolved *in vivo* by using appropriate voltage-reporters (see Fig. 4 of ref. 1 for voltage-sensitive fluorescent dyes revealing spatio-temporal patterns of bioelectrical gradients *in vivo*., Fig. 1 of ref. 31 for membrane potential gradients across the anterior-posterior axis of planarian flatworms, Fig. 6 of ref. 23 for a voltage reporter assay demonstrating long-term changes of the bioelectrical connectivity in octanol-exposed planaria, Figs 2 and 8 of ref. 24 for the experimental distribution of membrane potentials in *Drosophila* ovarian follicles, and ref. 25 for methods of visualising gap junction networks). The spatio-temporal regionalisation of cell polarisation is a crucial step to patterning and tumorigenesis because it regulates the transport of signalling molecules and ions such as serotonin, butyrate, and calcium which influence different biochemical pathways. Changes in the cell potential are usually transduced into downstream cascades and transcriptional processes (see Fig. 3b of ref. 1 for different mechanisms transducing modifications of the cell potential into downstream cascades and transcriptional changes).

The electrically-driven transport of signalling molecules through the intercellular gap junctions regulates the differences between the left and right sides in the *Xenopus* embryo (see Fig. 2 of ref. 27 for the case of morphogen electrophoresis through these junctions and Fig. 3 of ref. 32 for serotonin signaling in the patterning of the left-right axis in chick and frog embryos). Also, the transport to and accumulation in polarised cells of serotonin, together with the membrane potential regionalisation, is involved in ectopic eye innervation (see Fig. 6 of ref. 28 for the induction of nerve growth via modulation of host resting potential). In planaria, the action of depolarising ion channels and gap junctions results in the spatial map of electric potentials that regulate regeneration (see Fig. 4 of ref. 1 for the influence of bioelectrical signals on head regeneration). The membrane potential regulates also the calcium flux, intracellular concentration, and intercellular transport^33^. In turn, the calcium signaling influences gene expression^1^. Recently available experimental tools for monitoring and modifying membrane potentials have shown the complex feedback between bioelectrical and genetic signals^1^,^9^,^10^,^23^.

Bioelectric patterning has also been studied during oogenesis in *Drosophila* ovarian follicles using fluorescent indicators and inhibitors. The spatial distributions of membrane potentials, intracellular pH and specific membrane channel proteins modulate the observed developmental processes. Significant similarities between the membrane potential patterns and the spatial activity of voltage-gated calcium channels have been found, suggesting a mechanism for transducing bioelectric signals into cellular responses^24^.

The electric potential-dependent rates of serotonin influence also the transcriptional downstream responses observed in the metastatic behaviour of melanocytes (see Fig. 6 of ref. 14 for a mechanism transducing voltage changes into transcriptional cascades with the serotonin transporter SERT) and the electrically-driven transport of butyrate appears to be involved in tumour suppression in *Xenopus* embryos (see Fig. 8 of ref. 14 for the particular bioelectric mechanism). Intercellular communication^33^ is also crucial: a decreased gap junction activity is associated with the initial stages of some cancers in animal models and humans (see Fig. 1 of ref. 34 for the formation of induced tumour like structures that are gap junctionally connected to the host and refs 35 and 36 for the characterisation of defective gap junctional intercellular communication in carcinogenic processes). However, the role of gap junctions depends on the states of the neighbouring cells and the particular signals to be transferred and this context-dependent behaviour constitutes a problem for practical applications^35^,^37^.

## Genetic and Bioelectric Coupling

**Figure 1:**
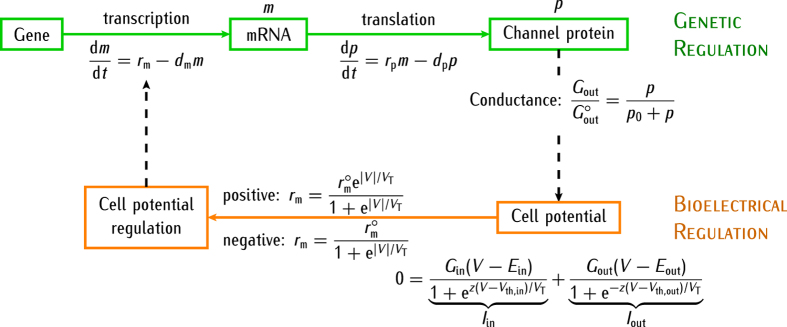
Scheme illustrating the positive and negative feedback scenarios between the genetic and bioelectrical descriptions at the single-cell level. The model equations for the time (*t*)-dependent coupling between the genetic and bioelectric regulations are also shown. The dashed arrows show the feedback mechanism between the potential-dependent production of the protein forming the outward-rectifying ion channel and the cell potential modulation due to this channel conductance. The transcription (*r*_m_) and translation (*r*_p_) rate constants together with their corresponding degradation rate constants *d*_m_ and *d*_p_ lead to the steady-state mRNA and protein concentrations *m* = *r*_m_/*d*_m_ and *p* = *r*_p_*m*/*d*_p_ = *r*_p_*r*_m_/(*d*_p_*d*_m_), respectively. For the sake of simplicity, the translation rate constant *r*_p_ and the degradation rate constants *d*_m_and *d*_p_ are assumed to be independent of the potential^38^,^39^,^40^. On the contrary, the transcription rate *r*_m_(*V*) depends on the cell potential *V* and can be increased (*positive* regulation) or decreased (*negative* regulation) with respect to the zero-voltage value 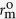 according to the absolute value |*V*|. The potential *V* is determined by the equation of zero total current through the membrane^29^, *I*_in_ + *I*_out_ = 0, where *I*_in_ and *I*_out_ are the electrical currents through the inward- and outward-rectifying channels^19^, respectively. The channel equilibrium potentials are fixed to *E*_out_ = 0 mV and *E*_in_ = −60 mV and the threshold potentials are *V*_th,in_ = *V*_th,out_ = −27 mV. The number of effective charges involved in gating^29^ is assumed to be *z* = 3 and the thermal potential is *V*_T_ = *RT*/*F* = 27 mV at 310 K, where *R* is the gas constant, *T* is the temperature, and *F* is the Faraday constant.

The above experimental facts suggest that multicellular electrical regionalisation should be coupled with local genetic regulation. Figure 1 shows a simple scheme illustrating the two feedback scenarios considered for the genetic and bioelectrical descriptions together with the relevant biophysical equations. The simplest modeling of genetic regulation is based on the mean-field equations^38^,^39^,^40^,^41^,^42^,^43^,^44^,^45^. As to the bioelectrical regulation, we assume that a particular ion channel in the cell membrane, the voltage-gated outward-rectifying channel, is formed by a specific protein whose concentration *p* depends on the intracellular concentration *m* of the corresponding mRNA. The conductance *G*_out_ regulated by this channel protein contributes to the total membrane conductance, thus allowing the modulation of the cell potential *V*. In turn, the channel protein concentration *p* in the cell is regulated by *V* because of the potential-dependent transcription assumed in Fig. 1. Note that we consider relative values for *p* and *m*, calculated with respect to a particular reference value, because the absolute concentrations should depend on the particular biochemical system considered.

The highly non-linear characteristics of the equations describing genetic^38^,^39^,^40^,^41^,^42^,^45^ and bioelectrical networks^19^,^22^,^29^ suggest significant feedback scenarios. The cell potential may influence the rate of gene transcription into mRNA, eventually resulting in changes of the rate of mRNA translation into proteins that should impact in the function of the protein ion channels. The effects of the membrane potential on gene expression are indirect and mediated by the following processes which are regulated by the potential difference *V* between the intracellular and extracellular media: (*i*) the calcium cell entry and subsequent regulation of genetic pathways^2^,^5^,^6^, (*ii*) the transport of different signaling molecules to the cell^1^,^2^,^3^,^7^,^41^, (*iii*) the electrically-induced conformational changes in the voltage-sensing domains of membrane proteins^1^,^4^, and (*iv*) the activation of voltage-gated potassium^5^,^46^,^47^ and calcium^2^,^6^,^24^,^48^,^49^ channels. These experimental facts constitute functional links between the genetic and bioelectric descriptions, establishing clear qualitative connections between the model and experimental results.

The existing experimental data for processes (*i*–*iv*) suggest an exponential dependence for *V* in most cases. Under quasi-equilibrium conditions, the intracellular concentrations of calcium and other positively charged molecules should increase exponentially with the absolute value of the negative cell potential |*V*|. Indeed, because the gradient of electric potential acts as a driving force for cell entry in processes (*i*) and (*ii*), the integration of the Nernst-Planck flux equation under quasi-equilibrium conditions gives an exponential dependence^29^ of the species concentration on the potential *V*. If we assume Hill kinetics for the dependence of the transcription process with the calcium concentration, the effective value of *r*_m_ in Fig. 1 should incorporate then the appropriate exponential functions for the cases of *positive* and *negative* regulation. Note also that the electrically-induced changes in the protein voltage-sensing domains (process (*iii*)) and the activation of voltage-gated channels (process (*iv*)) should also depend exponentially on the cell potential because of the respective Boltzmann factors that govern these processes^19^,^29^,^38^. Typically, the Boltzmann factors are exponentials of the electrical energy term, which includes the cell potential, divided by the thermal energy^29^.

Because the membrane ion channels controlling the potential *V* are proteins, they can be regulated by the protein concentration *p* of Fig. 1. This regulation is complex and may be due to structural changes such as phosphorylation, involving different spatial and time scales. In our phenomenological model, we incorporate the effect of *p* on the ion channel conductance by considering that the protein inserts into the cell membrane to form an outward-rectifying ion channel of conductance *G*_out_. This fact implicitly assumes that the ion channel response occurs on a time scale much faster than the gene expression, which is a realistic condition^38^,^39^,^40^. The above assumptions are simple and permit to introduce the genetic control in the bioelectric regulation of Fig. 1 using basic biophysical concepts.

Considering again a Hill kinetics scheme, the dimensionless conductance 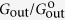 should follow the dependence on the protein concentration *p* shown in Fig. 1, where 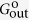 is the maximum value of this conductance and the reference concentration *p*_*0*_ corresponds to the conductance 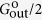. Note that *G*_out_ is an *output parameter* of the genetic regulation but an *input parameter* for the bioelectric regulation of Fig. 1 because the cell potential *V* is regulated by the outward- and inward-rectifying channels of conductances *G*_out_ and *G*_in_ in the bioelectrical model^19^ of Fig. 1.

## The single cell: Model Parameters and Results

There are many ion channels and pumps relevant for cell biology^22^,^29^,^50^. We consider only two generic voltage-gated channels because these channels are central to cell signalling^29^,^51^ and provide useful qualitative results with moderate mathematical complexity^19^,^52^,^53^,^54^,^55^. More complete computational models for the electrical conductance regulation of the resting membrane potential have also emphasised the dominant contributions of inward and outward-rectifying channels^51^,^53^. Increasing the complexity of the theoretical model by adding more channels and pumps should lead to additional feedback mechanisms that will invariably obscure the primary goal of this study: to show that bioelectrical phenomena occur at a different level than cell genetics and that the view of channel function as a direct gene product should be revised (see ref. 7 and Fig. 1 here).

Although multistability is inherent to many biological networks, membrane potential bistability^52^ is not a necessary condition for an efficient coupling between the genetic and bioelectrical descriptions but it allows a clear distinction between single-cell states in the spatial pattern of potentials^19^. It should be noted, however, that electrical bistability is crucial for the dynamics of neural cells and that long-term changes in the neuron function are influenced by the interplay of membrane potential pulses and gene expression^39^. Therefore, it is of biological interest to explore the consequences of bistability for the case of non-neural cells. Experimentally, bistability has been observed in lysenin channels inserted into lipid bilayer membranes^54^, the membranes of saccular hair cells in the green frog^55^, and skeletal and mouse lumbrical muscle cells^53^. Recently, resting potential bistability has been systematically studied in the modelling of a wide variety of amphibian embryos and mammalian cells using the *Channelpedia* ion channel database^56^. These biologically-oriented simulations emphasised the importance of voltage-gated channels for mammalian channels which can stably maintain one of several resting potentials^56^.

The inward-rectifying channel^19^,^29^ gives low outward currents for potentials *V* > *E*_in_, where *E*_in_ is the channel equilibrium potential, and large inward currents for *V* < *E*_in_. On the contrary, the outward-rectifying channel^19^,^29^ gives low inward currents for *V* < *E*_out_, where *E*_out_ is the channel equilibrium potential, and large outward currents and for *V* > *E*_out_. The potentials *E*_out_ and *E*_in_ are constant provided that the ionic concentrations are kept approximately constant in the extracellular environment and the intracellular solution^19^,^57^. Note in this context that the number of ions to be transferred in order to set up typical potential differences is very small compared with the total number of ions in the cell^58^.

**Figure 2:**
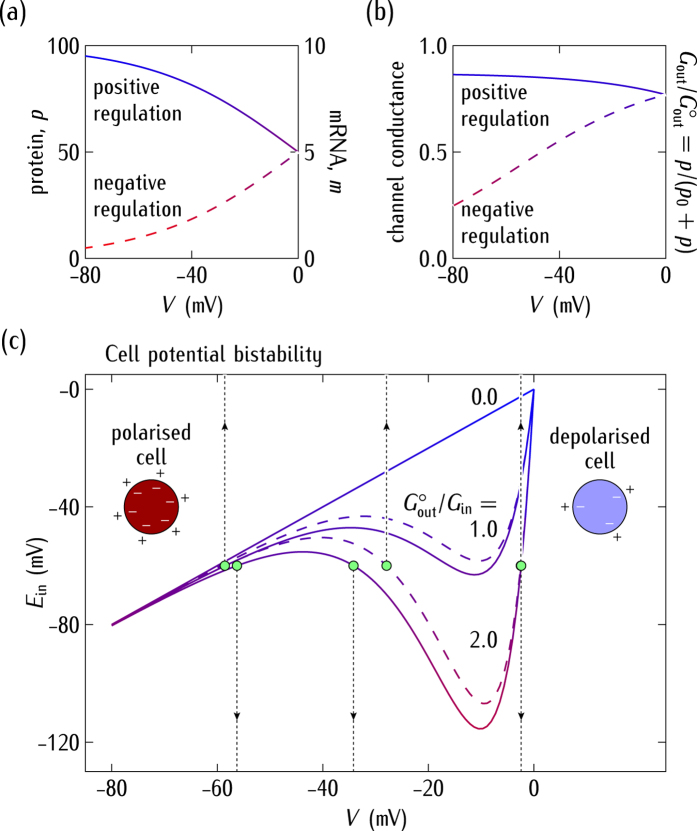
(**a**) The steady-state relative concentrations of mRNA (*m*) and protein (*p*) as a function of *V*. (**b**) The normalised outward conductance 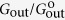 resulting from the values of *p* as a function of *V*. (**c**) The cell potential *V* obtained by solving the bioelectrical equation of Fig. 1 as a function of the equilibrium potential *E*_in_ parametrically in the outward to inward conductance ratio 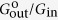. The cases of *positive* (continuous curves) and *negative* (dashed curves) regulation are considered. For particular values of *E*_in_ and 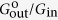, the cell potential enters into a bistability region where three (*polarised*, *unstable*, and *depolarised*) values for *V* are possible. The vertical arrows show these potentials for the reference case 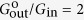 considered here.

Consider now the genetic regulation. The dynamics of mRNA transcription is complex and can be affected by heterogeneity effects^44^,^59^,^60^. The values of the rate constants in Fig. 1 are difficult to establish because the transcription, translation and degradation processes depend on multiple factors^38^,^39^,^42^,^43^,^44^,^61^. The transcription rate constants^38^,^42^,^43^,^61^ are in the range 0.1–5 min^−1^ while the translation rate constants^38^,^42^,^43^ are in the range 0.1–2 min^−1^. The time scales may vary between minutes and hours for mRNA and protein degradations, which gives rate constants^38^,^42^,^43^,^44^,^61^ in the range 0.003–0.1 min^−1^. We have considered the intermediate, biologically reasonable values 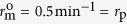 and *d*_m_ = 0.05 min^−1^ = *d*_p_ to obtain the steady-state values of the mRNA and protein relative concentrations of Fig. 2a. We introduce the reference value *p*_0_ = 15 in the equations of Fig. 1 to obtain the significant changes of the channel conductance *G*_out_ on the cell potential *V* of Fig. 2b.

The numerical solutions for *V* obtained from the cell potential equation of Fig. 1 are shown in Fig. 2c as a function of the equilibrium potential *E*_in_ for different conductance ratios 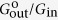. The solutions correspond to those points where the horizontal line *E*_in_ = constant intersects the different curves in the *E*_in_ − *V* plane. For some values of the system parameters, there are three numerical solutions corresponding to the *polarised* (high value of |*V*|) normal state, the *depolarised* (low value of |*V*|) abnormal state, and the *unstable* (intermediate value of |*V*|) state. The model cell acts as a *dynamical system* undergoing transitions between the above bioelectric states. These transitions are regulated by the relative contributions of the inward (cell polarising) and outward (cell depolarising) channels to the total membrane conductance^19^,^52^.

Figure 2c shows that an increase in the conductance ratio 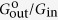 decouples *V* from the normal polarised value *E*_in_ allowing the cell potential to enter the bistability regime where abnormal depolarised values can be attained. Since the electrical conductance can be gated post-translationally^7^,^30^ e.g. by channel blocking^19^, the ratio 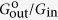 in Fig. 2c could be decreased externally, reverting the cell potential to the normal value. Alternatively, this effect could also be achieved by changing the external concentrations to shift the equilibrium potential^29^ *E*_in_, as shown in Fig. 2c. We note that up-regulation of sodium channels has been related to some types of cancer^62^,^63^,^64^. The up-regulation of the outward channels and down-regulation of the inward channels can be simulated by increasing the ratio 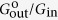 in the model^19^.

In summary, Fig. 2a–c show the *feedback scenario* between the cell potential and the protein forming the outward-rectifying ion channel. The value of |*V*| may increase (*positive* regulation) or decrease (*negative* regulation) the transcription of the mRNA that encodes this channel protein (Fig. 2a,b). In turn, the protein channel conductance can modulate the cell potential (Fig. 2c).

## The Multicellular Ensemble: Model Parameters and Results

**Figure 3:**
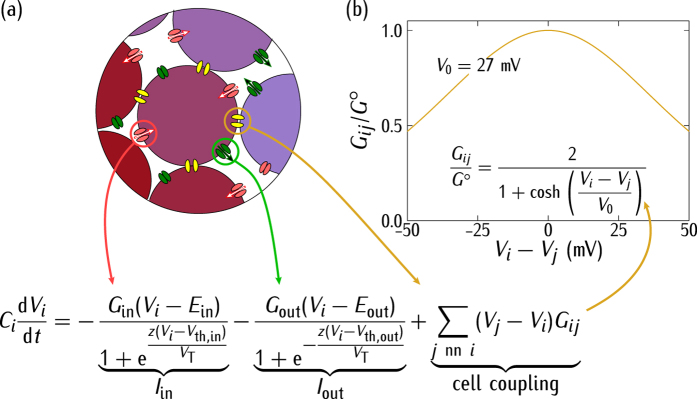
(**a**) The central cell *i* is coupled to the neighbouring cells *j* by protein gap junctions of conductance *G*_ij_. (**b**) The dimensionless conductance *G*_*ij*_/*G*^*o*^ follows a bell-shaped curve^35^,^36^,^66^,^67^ as a function of the potential difference *V*_*i*_ − *V*_*j*_ between two adjacent cells *i* and *j*. The curve is characterised by the maximum gap junction conductance *G*^*o*^/*G*_in_ = 0.5 and the width potential *V*_0_ = 27 mV. In the model equation, the time evolution of the potential *V*_*i*_ depends on the single-cell currents *I*_in_ and *I*_out_ of Fig. 1 and the intercellular coupling current, which is regulated by *V*_*i*_ − *V*_*j*_. The subscript *j* refers to summations restricted to the cell nearest neighbours (nn) and the cell capacitance *C*_*i*_ is considered to be the same for all cells. The equation for *V*_*i*_ allows extending the genetic and bioelectric models from the single-cell level of Fig. 1 to the multicellular level.

The cell is not isolated but connected to other cells in a multicellular system^65^. Bioelectrical signals extend beyond the single-cell states because of the intercellular channels (gap junctions) that allow the transfer of electric currents and signaling molecules between neighbouring cells. Decreased intercellular communication enhances autonomous cell behaviour and has been related to tumorigenesis^36^,^37^,^65^. We assume that because of the low frequency signals characteristic of non-excitable cells, the conductive coupling dominates over the capacitance coupling^19^. Figure 3a,b show the intercellular coupling model together with the relevant biophysical equations that permit multicellular electrical states. The coupling is accounted for by effective gap junctions of conductance *G*_*ij*_ which can be due to the connexin protein family^35^,^36^,^66^,^67^. Despite the high the number of gap junctions per cell, only a very small fraction of open channels participate effectively in intercellular coupling^27^,^56^,^68^. We assume that the effective gap junction conductance per cell is of the same order of magnitude as the total cell conductance due to the inward and outward-rectifying channels. This condition assures that the two conductances are relevant in the model equation of Fig. 3b. Note that too low gap junction conductances would give essentially isolated cells while too high values of these intercellular conductances would produce an isopotential ensemble with no spatial regionalisation. The introduction of the dimensionless conductances 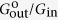 (Fig. 2c) and *G*^*o*^/*G*_in_(caption of Fig. 3b) allows showing the qualitative trends of the model by comparing the relative values of the gap junction and ion channel conductances.

We consider a system of *N* = 2524 cells initially at potentials *V*_*i*_(*t* = 0), *i* = 1, …, *N*. The initial concentrations of protein *p*_*i*_(0) and mRNA *m*_*i*_(0) are those obtained from the steady-state solution of the genetic regulation equations (see caption of Fig. 1) for *V*_*i*_(0). Then, we let the system to evolve with time, solving the *N* equations of Fig. 3a for *V*_*i*_(*t*) under the different conditions considered in each case study. The time evolution of these single-cell potentials induces changes in the transcription rate constant *r*_*m*_ of cell *i* (see Fig. 1). The feedback mechanism of Fig. 1 couples the genetic and bioelectric changes over the multicellular system because the outward-rectifying channel conductance depends on the cell protein concentration (Fig. 2a,b).

To better understand the ensemble dynamics, we note that there are *two characteristic times* in the single-cell and multicellular schemes of Fig. 1 and 3. The electrical relaxation is fast because of its relatively small characteristic time^13^ *C*_*i*_/*G*_in_ = 0.1 s for *G*_in_ = 1 nS and *C*_i_ = 100 pF. On the contrary, transcription and translation rate constants in the range 0.1–1 min^−1^ give characteristic times between 1 and 10 min while degradation rates constants in the range 0.003–0.1 min^−1^ give times between 0.1 and 5 h^38^,^42^,^43^,^57^. Diffusion is not incorporated in the model, but it should give a characteristic time *L*^2^/*D* greater than 1 h for an ionic diffusion coefficient *D* = 10^−10 ^m^2^/s and a distance *L* = 10^−3 ^m, which corresponds to about 100 cell diameters^19^. Thus, the longer characteristic time is that of the genetic regulation if diffusion processes are ignored. These processes may significantly increase the time response if they are incorporated in the model^19^,^20^,^22^. Recently, a finite volume method simulator accounting for diffusion and ionic concentration changes has been proposed by Pietak and Levin to study the spatio-temporal dynamics of bioelectric patterns^22^.

Genetic processes occur on a time scale much longer than electrical relaxations for the biologically plausible parameters introduced here. The significantly different time scales obtained suggest the following procedure for the system simulation. A single-cell in the ensemble should be initially in one of the bioelectric states of Fig. 2c determined by the value of *V* and the protein concentration *p* in Fig. 2a,b. Because of the coupling provided by the gap junction conductances of Fig. 3a, the other cells can rapidly change the cell potential *V* to a new value and then the protein concentration *p* will relax slowly to a new value compatible with this potential. Since the electrical relaxation is much faster than the genetic relaxation, we can obtain first the steady-state values of *V* and take them as constants in the calculation of the *p* values. This procedure allows formally decoupling the time-dependent equations for the bioelectrical and genetic regulations of Fig. 1.

**Figure 4:**
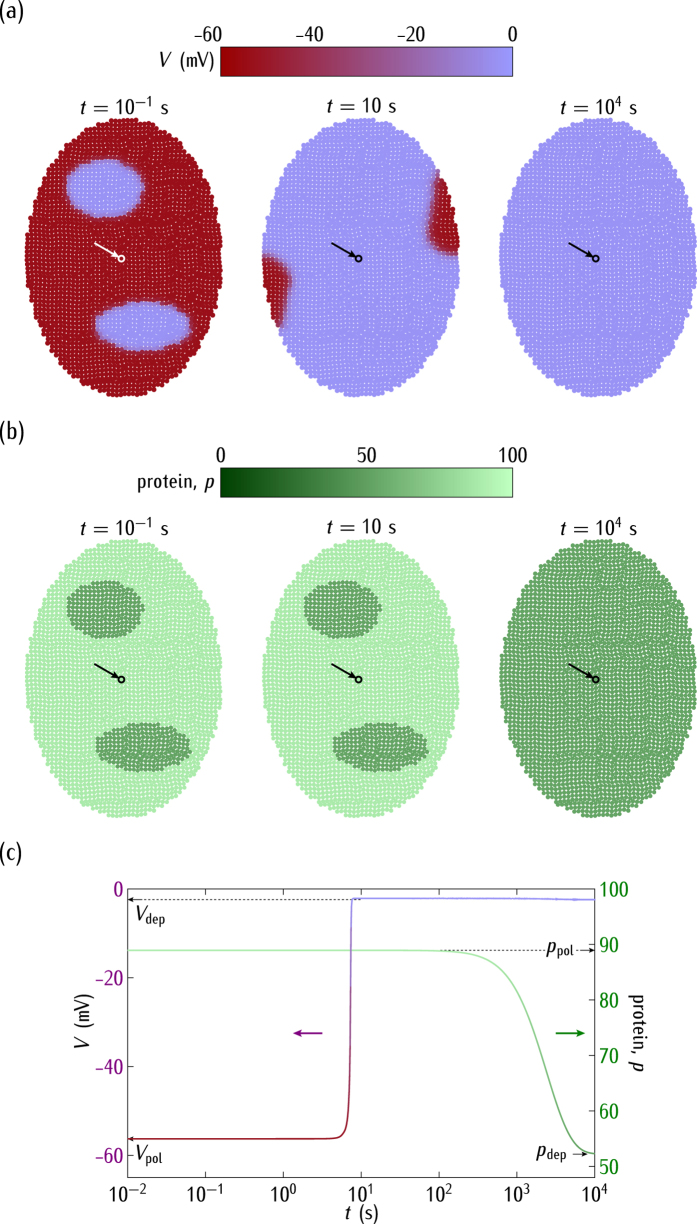
(**a**) The cell potential spatial regionalisation. (**b**) The protein regionalisation. (**c**) The time relaxations of *V* and *p* for the central cell marked by the arrow. We consider the *positive* regulation case of Fig. 1 with 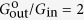 in Fig. 2c. The horizontal bars are the scales of *V* and *p*. Initially, the cell potentials are locally depolarised (*V* = −2.4 mV) at the two small regions while they are polarised (*V* = −59 mV) in the rest of the system. The rate and degradation constants are those of Fig. 2. At short times, the electrical relaxation gives a predominantly depolarised ensemble. At long times, the genetic relaxation results in low values of the protein concentration. The subscripts *pol* and *dep* make reference to the polarised and depolarised values, respectively.

Consider now the ensemble dynamics for different case studies. At time *t* = 0, the cell potentials in the two small patches of Fig. 4a are in a region corresponding to the depolarised potential in Fig. 2c while the rest of the cells in the system have a polarised potential. For the case of *positive* regulation in Fig. 1, the outward-rectifying channel conductance is high (Fig. 2b) and then the *depolarised* cell state is dominant in the bistability region of Fig. 2c. Thus, the cell electrical coupling forces the depolarised state over the whole ensemble of Fig. 4a after a short time. This effect shows clearly the *ensemble nature* of the electric potential in this model because the final isopotential state is a consequence of the concerted collective behaviour^30^.

Due to the *positive* regulation and the low single-cell potentials attained in Fig. 4a, the genetic relaxation gives low values of the protein concentration at sufficiently long times (Fig. 4b; see Fig. 2a for the correspondence between *p* and *V*). As expected, the electrical relaxation is fast compared with the protein concentration changes, as shown in Fig. 4c for the time dependence of *V* and *p*.

**Figure 5:**
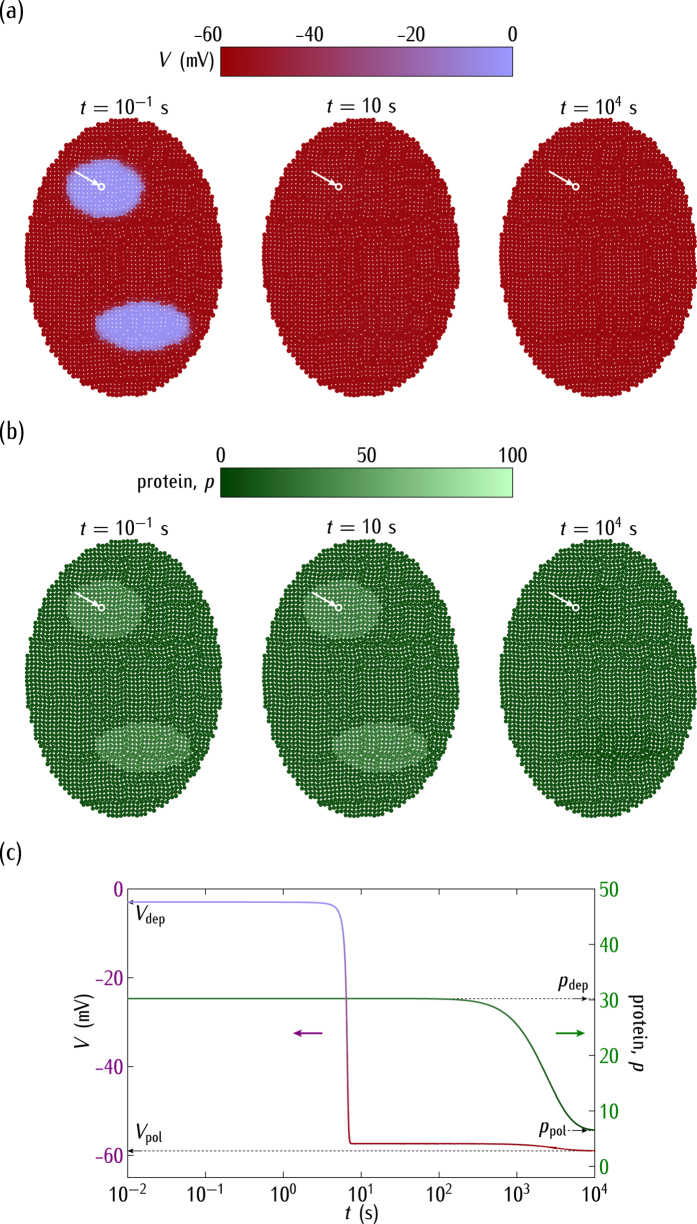
(**a**) The cell potential spatial regionalisation. (**b**) The protein regionalisation. (**c**) The time relaxations of *V* and *p* for the central cell marked by the arrow. We consider now the case of the *negative* regulation in Fig. 1. As in Fig. 4, the cell potentials are initially depolarised (*V* = −3 mV) at the two predefined regions where 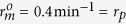 and polarised (−59 mV) in the rest of the system characterised by the reference rate constants 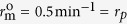 of Fig. 2. Because of the *negative* regulation, the electrical relaxation gives a polarised ensemble (short times) while the genetic relaxation gives low protein concentrations (long times).

Figure 5a considers the case of *negative* regulation and *different* genetic characteristics between the two small regions and the rest of the system. As in Fig. 4a, the cell potentials are initially depolarised in these regions while they are polarised in the rest of the ensemble. For the case of the *negative* regulation, however, it is the *polarised* cell state that is dominant (Fig. 5a) rather than the depolarised one (Fig. 4a). Thus, the electrical relaxation gives now a polarised system after a short time. The subsequent genetic relaxation gives again very low values of the protein concentration (Fig. 5b,c) because of the *negative* regulation and the high values of |*V*| attained by the single-cell potentials (see Fig. 2a). Figure 5a,b show that a slightly different genetic pre-pattern established over adjacent spatial regions can be smoothed out by bioelectrical signals, which suggest that endogenous bioelectrical networks may influence patterning during development and regeneration^7^. Note also that gene expression is intrinsically probabilistic^43^,^45^ and Fig. 5a,b suggest that the intercellular coupling may favour the stabilization of this expression over adjacent spatial regions.

**Figure 6:**
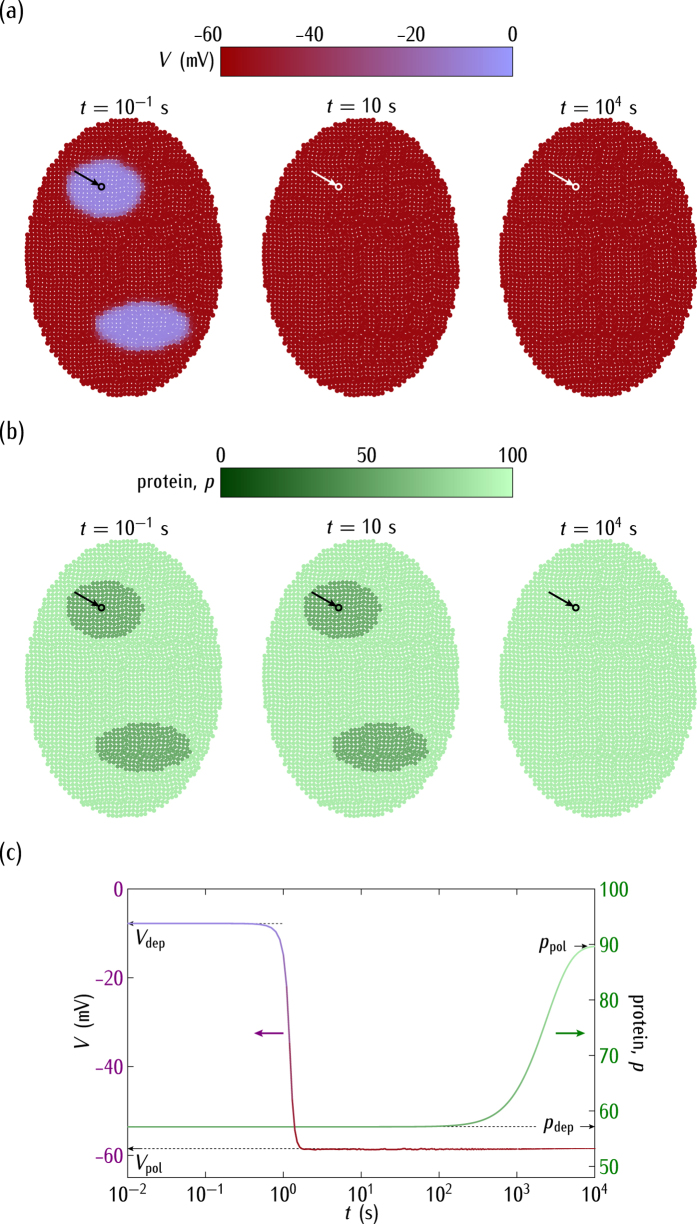
(**a**) The cell potential spatial regionalisation. (**b**) The protein regionalisation. (**c**) The time relaxations of *V* and *p* for the central cell marked by the arrow. As in Fig. 4, we consider the case of the *positive* regulation, but now for the decreased conductance ratio 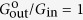, which gives values of |*V*| higher than those of Fig. 4. Initially the cell potentials are depolarised (*V* = −8 mV) at the two small regions while the rest of the system is polarised (*V* = −59 mV). All over the system, we use the reference rate constants 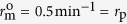. The electrical relaxation gives a predominantly polarised ensemble. The genetic relaxation results in high values of the protein concentration because of the *positive* regulation.

Figure 6a considers again the *positive* regulation of Fig. 4a but now we assume 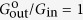 instead of 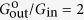, which leads to the prevalence of the *polarised* rather than the *depolarised* state (see Fig. 2c; compare Fig. 6a with Fig. 4a). This decreased conductance ratio is assumed to occur *post-translationally* and gives values of |*V*| higher than those of Fig. 4a, as it should be expected from Fig. 2c. Initially, the cell potentials are depolarised within the two small regions while the rest of the system is polarised. The electrical relaxation results now in a polarised ensemble that supports high values of the protein concentration at long times (Fig. 6b,c) because of the *positive* regulation.

Note the different protein regionalisations which are obtained in Figs 4b and 6b despite the fact that the genetic map of rate constants is the same in these figures. This result shows that the integration of bioelectric circuits with transcriptional regulation may have implications for multicellular patterning and regeneration. In particular, Figs 4b and 6b suggest that *distinct bioelectric states* resulting from *different post-translational gating* of the conductance ratio 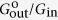 can *override identical genetic pre-patterns*. This conclusion is relevant for the developmental and regeneration problems described in ref. 7 concerning endogenous bioelectrical networks.

**Figure 7:**
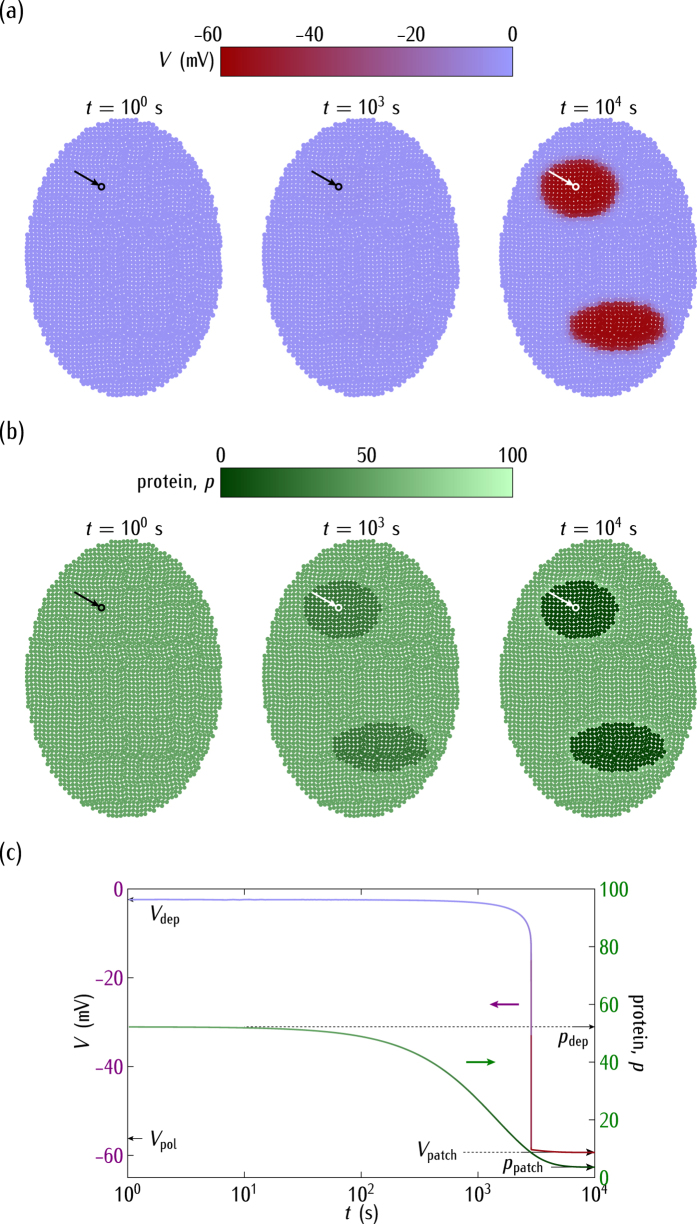
(**a**) The cell potential spatial regionalisation. (**b**) The protein regionalisation. (**c**) The time relaxations of *V* and *p* for the central cell marked by the arrow. The cells are initially in the depolarised state (*V* = −2.4 mV) of Fig. 4a but they are subject now to *negative* regulation. We assume that the rate constants in the two small regions shift to the decreased values 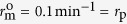 at *t* = 0 while the reference values 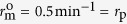 prevail in the rest of the system. These regions evolve then towards new values of the polarised potential (*V* = −59 mV) and low protein concentration that are compatible with their locally different rate constants.

Figures 5 and 6 show that regions of cells initially in different genetic and bioelectric states can eventually reach the same isopotential state. Figure 7 considers the *opposite* case: a system of cells initially at the same potential can eventually undergo an electrical regionalisation (Fig. 7a) because of the genetic differences between adjacent spatial regions (Fig. 7b). In this case, it is the genetic regulation change that precedes the electrical regionalisation (Fig. 7c). Note also that the depolarised cells are not able now to impose their electrical state over the whole ensemble because the genetic differences lead to distinct potentials at the interfacial regions, which decreases the voltage-dependent connectivity, as shown in Fig. 3b. In all the cases studied, the dynamics of the single-cell potential (Fig. 2c) together with the intercellular voltage-gated gap junctions (Fig. 3a) determine the cell bioelectric states which are coupled with the corresponding genetic states (Fig. 1).

Interestingly, *shifts in the normal cell electrical state could trigger significant changes in the genetic regulation*. The complex interplay between bioelectrical and genetic signals has been noted in different experimental contexts^1^,^2^,^5^,^7^,^9^,^10^. In particular, Fig. 4 shows the ensemble nature of the electrical potential^19^,^30^, Fig. 5 suggests that bioelectric signals could override slightly different genetic pre-patterns^1^,^7^,^30^, Fig. 6 emphasises the importance of post-translational gating^7^,^30^, and Fig. 7 describes a mechanism by which an ensemble of cells initially at the same potential can undergo an electrical regionalisation because of persistent genetic differences between adjacent regions.

The single cell-centred approach emphasises channels as specific proteins that determine individual cell properties, thus disregarding their contribution to the multicellular outcomes of Figs 4, 5, 6 and 7. We have shown here that heterogeneous multicellular patterns can be obtained by altering the electrical balance between the ion channels in the cell membranes over local regions of the system^19^. Obviously, the particular ion channels and pumps involved for each biological cell should be different^1^,^2^,^5^,^29^,^56^ but the present model provides useful qualitative insights with only two generic voltage-gated channels. While intercellular diffusional processes leading to response times longer than those found here should be incorporated in the model^19^,^22^, the results suggest that the interplay between the bioelectric and genetic regulations can certainly play a role in the establishment of spatial patterns in multicellular systems. In particular, the conceptual scheme of Figs 4, 5, 6 and 7 should be useful for the modelling of left-right patterning^22^,^32^ where bioelectrical asymmetry can eventually result in asymmetric gene expression. Also, extensions of the model equations to include additional feedback scenarios may have qualitative implications for other experimental problems where cell potentials are involved^1^,^14^,^16^,^17^,^18^,^25^,^30^.

**Figure 8:**
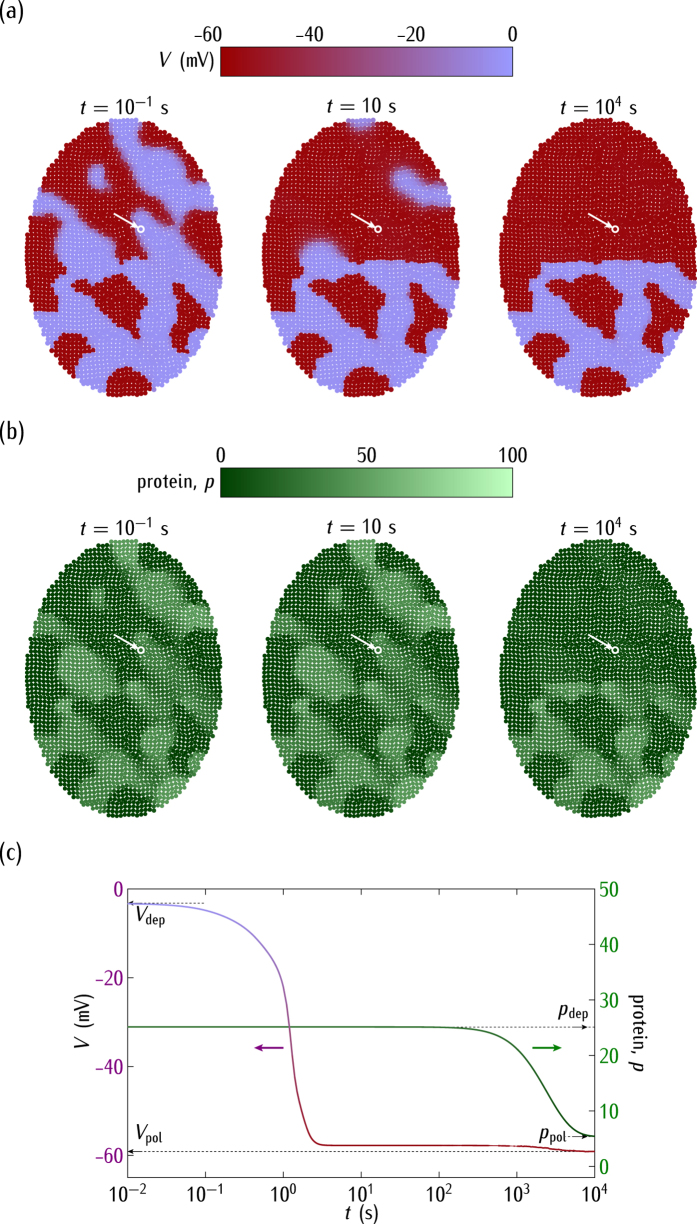
(**a**) The cell potential regionalisation caused by a spatial heterogeneity of rate constants and gap junctions. (**b**) The protein regionalisation. (**c**) The time relaxations of *V* and *p* for the central cell marked by the arrow. We consider the *negative* regulation case of Figs 5 and 7, with 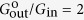 in Fig. 2c. The multicellular ensemble is divided in heterogeneous spatial regions with different genetic pre-patterns. The rate constants 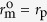 take values between 0.1 and 0.2 min^−1^ in the darker regions of low protein concentration while they are between 0.4 and 0.5 min^−1^ in the lighter regions of high protein population. The degradation rate constants are those of Fig. 2. The coupling gap junction conductance is *G*^*o*^/*G*_in_ = 0.5 in the top half of the ensemble while it decreases to *G*^*o*^/*G*_in_ = 0.05 in the bottom half. Initially, the cells in the darker regions are in a monostable state with a polarised potential while the cells in the lighter regions are in a bistable state with a depolarised potential.

Most biological ensembles show a significant individual diversity and we consider finally the problem of heterogeneity within the cell population. To this end, we have introduced statistical distributions in Fig. 8 for the rate constants 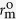 and *r*_p_ which take values between 0.1 and 0.2 min^−1^ in the darker regions of low protein concentration whereas these constants are between 0.4 and 0.5 min^−1^ in the lighter regions of high protein population. To better show the interplay between the genetic and bioelectrical regulations, this heterogeneity coexists with a spatial distribution of gap junction conductance values which are 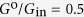 in the top half of the ensemble but 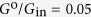 in the bottom half.

Figure 8 shows that the heterogeneous prepatterns of rate constants and coupling conductance values increase the dynamic range of the system response, allowing complex regionalisations in the multicellular ensemble^1^,^7^,^23^. In particular, the results suggest that a spatially heterogeneous coupling between bioelectric and genetic signals should drive distinct biological outcomes: a quasi-homogenous potential in the top of the ensemble coexists with a heterogeneous distribution of potentials in the bottom. Because it is experimentally possible to establish a partial disruption of the intercellular connectivity and identify spatially different bioelectrical and genetic patterns^23^, Fig. 8 provides qualitative insights into experimental studies on electrical and genetic regionalisations^1^,^7^. Note also that the different isopotential domains of Fig. 8 should produce a heterogeneous map of local concentrations (see Fig. 8 of ref. 19) for charged signalling molecules which are relevant to embryogenesis and regeneration processes^1^,^24^,^27^.

## Conclusions

There are solid experimental evidences relating genetic regulation and cell electrical polarisation^1^,^2^,^5^,^9^,^10^,^11^,^46^,^47^. Because ion channel and gap junction proteins are regulated by the cell potential and in turn regulate this potential, it is of interest to study the interplay between bioelectrical signaling and gene regulatory networks in multicellular processes such as patterning. Because of the inherent non-linearity of genetic and bioelectrical processes, complex feedback scenarios arise from the spatio-temporal integration of the corresponding networks. In this biological context, simple models aimed at understanding the emerging dynamics should be useful. Note also the potential biomedical opportunities that may offer a qualitative understanding of the basic concepts involved for reprogramming cells and tissue patterning via bioelectrical pathways^1^,^10^.

The single-cell genetic regulation considered here is based on mean-field kinetic equations for the mRNA and protein relative concentrations. These equations could now be extended to more complex genetic networks^41^,^45^ and coupled to the bioelectrical description of Fig. 1. In our case, the transcription is assumed to depend on the cell potential, which is modulated by the voltage-gated cell ion channels and the intercellular gap junctions. This theoretical approach constitutes *a preliminary attempt to integrate different regulation pathways, from the local cell level to the long range multicellular system.* In particular, we describe simple physical mechanisms to translate single-cell bioelectric states into multicellular regionalisations that can be observed using voltage-sensitive dyes^23^,^24^,^25^. While the importance of chemical signals and concentrations gradients in the genetic regulation and establishment of biological patterns is well documented^26^, the regionalisation of single-cell polarisations is not well understood and contributes also to the spatial distribution of signalling molecules in the ensemble^1^,^7^,^23^,^28^.

In conclusion, Figs 4, 5, 6, 7 and 8 show that the bioelectric state of the multicellular ensemble may not be uniquely determined by the underlying transcriptional state because of the feedback mechanisms and the fact that ion channels and gap junctions can be gated post-translationally, as noted in previous experimental work^1^,^18^. The local sensing ability of a single-cell should not be sufficient to develop and maintain efficiently complex biological patterns^1^. *Multicellular phenomena could achieve a spatially distributed control by the conversion of local genetic and electric processes into long range effects using intercellular coupling*. This conversion may involve not only reaction-diffusion processes but also bioelectrical signals and genetic regulation because the resulting coupling may provide different multistability and feedback scenarios ideally suited to establish long-term memory patterns^1^,^7^,^19^.

## Additional Information

**How to cite this article**: Cervera, J. *et al*. The interplay between genetic and bioelectrical signaling permits a spatial regionalisation of membrane potentials in model multicellular ensembles. *Sci. Rep.* **6**, 35201; doi: 10.1038/srep35201 (2016).
